# Vietnamese Consensus on the Structure and Content of Asthma Action Plan

**DOI:** 10.3390/jcm14248640

**Published:** 2025-12-05

**Authors:** Quan Vu Tran Thien, Tho Nguyen Van, Quyen Thi Le Pham, Linh Duong Thi Chuc, Cong Nguyen Hai, Tuan Tran Trong Anh, Khai Ho Quoc, Huong Hoang Thi Lan, Lan Le Thi Tuyet

**Affiliations:** 1 Department of Physiology—Pathophysiology—Immunology—Pharmacology, School of Medicine, University of Medicine and Pharmacy at Ho Chi Minh City, Ho Chi Minh City 72721, Vietnam; 2 Department of Respiratory Functional Exploration, University Medical Center Ho Chi Minh City, Ho Chi Minh City 72721, Vietnam; 3 Department of Tuberculosis and Lung Diseases, School of Medicine, University of Medicine and Pharmacy at Ho Chi Minh City, Ho Chi Minh City 72721, Vietnam; 4 Respiratory Department, Tam Anh Hospital, Ha Noi 11813, Vietnam; phamlequyenbmh@gmail.com; 5 Kien Giang General Hospital, An Giang 91118, Vietnam; 6 Department of Respiratory Medicine, Military Hospital 175, Ho Chi Minh City 71423, Vietnam; nguyen_med@ymail.com; 7 General Medicine Department, Can Tho University of Medicine and Pharmacy Hospital, Can Tho 94120, Vietnam; ttatuan.bv@ctump.edu.vn; 8 Gia Dinh People’s Hospital, Ho Chi Minh City 72330, Vietnam; 9 Hue Central Hospital, Hue 49135, Vietnam

**Keywords:** asthma action plan, Delphi consensus, agreement, self-management, asthma

## Abstract

**Background/Objectives**: Asthma action plans (AAPs) are recommended for patients’ self-management of asthma and should be adapted to a country’s situation. This study aimed to develop expert consensus on the optimal structure, content, and action of asthma action plans for Vietnamese settings to ensure feasibility, acceptance, and implementation. **Methods**: A Delphi consensus was conducted over two rounds. The proposed items were evaluated by a Vietnamese panel of pulmonologists, allergists, tuberculosis/lung disease specialists, and general practitioners. Structured online questionnaires with five-point Likert scales were used. Consensus was defined as >80% agreement and <10% strong disagreement. **Results**: A total of 26 and 21 participants completed round 1 and round 2, respectively. The 4-zone format of AAP was preferred (42.3%) over the 3-zone (38.5%) or 2-zone (19.2%) formats. The AAP should include some key statements for asthma, symptoms for self-monitoring, an objective asthma control questionnaire, actions for changes in maintenance medication, and instructions in emergency situations. AAP zones should be classified by symptom frequency and severity. Patient actions should be tailored to their treatment regimen (MART or ICS/LABA + SABA). The APP might not include peak expiratory flow monitoring and oral corticosteroid self-administration for both the MART and ICS/LABA + SABA regimens and might not add SABA together with ICS dose escalation for the ICS/LABA + SABA regimen. **Conclusions**: This study established an expert consensus on fundamental AAP structural elements and actions for the Vietnamese. The failure to achieve consensus on PEF monitoring tools and OCS for the self-management of asthma exacerbation reflects concerns about medication abuse, especially in Vietnamese healthcare settings.

## 1. Introduction

Asthma action plans (AAPs) are evidence-based tools designed to empower patients with asthma to recognize deteriorating symptoms, implement appropriate self-management strategies, and seek timely medical care when necessary [1,2]. These written, individualized plans have demonstrated significant clinical benefits, including reduced hospitalizations, emergency department visits, and improved symptom control when implemented alongside comprehensive patient education and regular follow-up [1,3]. Despite widespread endorsement by international asthma guidelines, including the Global Initiative for Asthma (GINA) and national guidelines worldwide, the structure and content of AAPs remain remarkably heterogeneous across healthcare settings, regions, and institutions [4,5,6].

The structural components of AAPs also demonstrate considerable variation. While evidence from randomized trials supports the use of 2–4 action zones, such as the green–yellow–red zone system, in individualized plans to consistently improve asthma outcomes [1], there remains no consensus on the optimal number of zones (e.g., two-zone versus three-zone systems), the specific symptom lists that maximize sensitivity and specificity for detecting exacerbations, or standardized action phrasing applicable across diverse healthcare settings [7]. This lack of standardization may contribute to confusion among patients and healthcare providers, potentially reducing the effectiveness of these critical self-management tools.

Asthma is a significant health challenge in Vietnam, with a prevalence of 3.9% [8]; research indicated that 69.6% of asthma patients seeking care at a tertiary medical facility had uncontrolled asthma [6]. The Vietnamese asthma patients also exhibited limited health literacy, partially managed symptoms, and insufficient adherence to prescribed medication [9]. The management of asthma by healthcare professionals constitutes a significant concern. In a survey regarding the therapeutic approaches for asthma, 83.1% primary care physicians in Vietnam prescribed oral steroids, 71.2% prescribed oral short-acting beta agonists, and 70% prescribed long-acting beta agonists without inhaled corticosteroids [10]. Recently, in 2025, the Ministry of Health of Vietnam amended the regulations to permit individuals with asthma to have a maximum interval of three months for follow-up consultations instead of one month, as previously mandated. An extended follow-up interval may elicit apprehensions regarding patients’ asthma management and medication compliance, and there ought to be a tailored instrument for their self-management.

There are various asthma action plan templates around the world [11,12,13], but patients from different ethnic groups benefit most from self-management materials that are tailored to the local culture, socio-economic situations, and availability of medication, rather than from generic tools [14]. In Vietnam, although recommendations regarding AAPs have been issued, most do not provide comprehensive information on asthma action plans [5,8] or use AAPs that have not been adapted to local contexts. Some components of international AAPs such as the self-administration of oral corticosteroid might not be suitable for Vietnamese circumstances. Therefore, it is essential to reach a consensus on developing an asthma action plan that is appropriate and practical for the Vietnamese context. The consensus might resolve concerns about the feasibility of applying AAPs developed in other countries and provide a customized, standardized instrument for both physicians and patients with asthma in Vietnam.

### 1.1. Consensus Development in Asthma Management

The Delphi method has emerged as a valuable approach for developing expert consensus in asthma management, particularly in areas where high-quality randomized controlled trial evidence is limited or where operational details require professional judgment [15]. This structured consensus technique has been successfully employed in various asthma-related contexts, demonstrating both feasibility and clinical utility.

Recent applications of the Delphi methodology in asthma include the development of national standards for severe asthma management [16,17], short-acting beta-agonist (SABA) use [18], or the Spanish asthma guidelines [19]. These studies demonstrate that the Delphi methodology can effectively bridge evidence gaps in asthma management, particularly when involving multidisciplinary panels of clinicians, educators, and other stakeholders. The success of these consensus efforts provides a strong foundation for applying a similar methodology to standardize AAP structure and content.

### 1.2. Study Objectives and Research Questions

This study aims to address the identified evidence gaps through systematic expert consensus development using a modified Delphi approach. The primary objectives are as follows:

1. Establish expert consensus on the ideal number of zones in an asthma action plan.
2. Identify a core set of symptoms and indicators for assessing asthma control.
3. Develop a standardized framework for classifying symptom severity within each zone.
4. Propose corresponding patient actions for each zone and severity level.

### 1.3. The Specific Research Questions Guiding This Investigation Are as Follows

1. How many zones or sections should an asthma action plan include to guide patients effectively?
2. What symptoms should be included in an asthma action plan to help patients recognize their asthma status?
3. How should symptom severity be categorized within each zone or section of the action plan?
4. What specific patient actions should correspond to each symptom or level of symptom severity?

By addressing these fundamental questions through structured expert consensus, this study aims to provide evidence-informed recommendations for standardizing AAP structure and content, ultimately improving the consistency and effectiveness of asthma self-management tools across healthcare settings.

## 2. Materials and Methods

### 2.1. Study Design

This study employed a Delphi methodology to develop expert consensus on the optimal structure and content of asthma action plans [20,21]. The survey was carried out from March to May 2025. The Delphi design incorporated structured online questionnaires administered over 2 rounds, with responses analyzed quantitatively to determine consensus achievement. The modification involved providing structured response options rather than completely open-ended questions in the initial round, which enhances efficiency while maintaining the iterative feedback process essential to Delphi methodology [22].

### 2.2. Expert Panel Selection

Inclusion Criteria: Expert panelists were selected based on the following inclusion criteria:

- Minimum of 5 years of clinical or research experience in asthma management.
- Healthcare professionals from relevant specialties, including pulmonologists, general practitioners with respiratory medicine expertise, specialists in tuberculosis and lung diseases, and allergists.
- Active clinical practice or research involvement in asthma care.

#### Sample Size and Sampling Strategy

The target sample size was 20–30 experts, determined based on established recommendations for Delphi studies in healthcare [23]. This sample size balances the need for diverse expert perspectives with the practical considerations of achieving meaningful consensus and maintaining manageable group dynamics throughout multiple rounds.

Purposive sampling was employed to ensure comprehensive representation across multiple dimensions:

- Geographical diversity: Experts from different regions of Vietnam to capture regional variations in clinical practice and healthcare delivery.
- Disciplinary diversity: Representation from pulmonology, general practice, and tuberculosis/lung disease specialties.
- Institutional diversity: Inclusion of experts from both public and private practice settings.
- Experience levels: Mix of senior clinicians and emerging experts to balance established wisdom with contemporary perspectives.

### 2.3. Recruitment Process: Expert Identification and Recruitment Followed a Systematic Approach

1. Professional Identification: Potential experts were identified through leadership positions in relevant hospitals in Vietnam.
2. Snowball Sampling: Initial identified experts were asked to recommend additional qualified colleagues who met the inclusion criteria, expanding the potential participant pool.
3. Invitation Process: Eligible experts received personalized email invitations that included the study background and objectives, the expected time commitment and timeline, an explanation of the Delphi methodology, and a link to the online survey platform.
4. Follow-up Protocol: Non-responders received up to two follow-up reminders at one-week intervals to maximize participation rates.

### 2.4. Delphi Procedure

Round 1:

The initial round presented experts with structured questionnaires covering core components of asthma action plan design. Participants were asked to rate their agreement with proposed items using a five-point Likert scale (Strongly Disagree, Disagree, Neutral, Agree, Strongly Agree) and provide qualitative comments for each section.

**Content Areas Addressed:**

- Zone Structure: Evaluation of optimal number of action plan zones (2-zone, 3-zone, or 4-zone systems) with rationale for preferences.
- Symptom Selection: Rating of proposed symptoms for inclusion in each zone, including both subjective symptoms (shortness of breath, wheezing, and chest tightness) and functional indicators (activity limitation, sleep disruption, and reliever medication use).
- Severity Definitions: Assessment of draft definitions for mild, moderate, and severe symptom categories within each zone.
- Patient Actions: Evaluation of proposed patient responses corresponding to each zone and severity level, including medication adjustments, activity modifications, and healthcare-seeking behaviors.
- Additional Components: Rating of supplementary elements such as PEF monitoring, emergency contact information, and educational content.
- Open-Ended Components: Each section included open-ended questions allowing experts to suggest additional symptoms, actions, or modifications to the proposed content. This qualitative input was essential for capturing expert knowledge not reflected in the structured items.

Round 2: Feedback and Re-Rating Phase

Individual Feedback: Each participant received their responses via email.

Group Feedback: Percentage agreement for each item from Round 1, and an anonymized summary of qualitative comments and suggestions from all participants.

Re-Rating Process: Participant were asked to reconsider their initial ratings considering the group feedback and either confirm their original positions or modify their responses. The questionnaire maintained the same five-point Likert scale format, and new items suggested during Round 1 were not included.

### 2.5. Consensus Definition and Criteria

Consensus achievement was defined using dual criteria adapted from established Delphi methodology guidelines [24,25]:

Consensus Achieved: An item was considered to have reached agreement when both of the following conditions were met: if more than 80% of the participants selected “Agree” or “Strongly Agree” and less than 10% of the participants selected “Strongly Disagree”. Percentage agreement was calculated by summing the percentages of participants who selected “Agree” and “Strongly Agree”.

These thresholds were selected based on a systematic review of the literature indicating that ≥80% agreement is widely recognized as a robust criterion for consensus in healthcare-related Delphi studies, while the ≤10% strong disagreement threshold ensures that consensus is not declared in the presence of substantial expert opposition [26]. The dual criteria approach prevents artificial consensus achievement when high neutral responses mask underlying disagreement.

### 2.6. Ethical Considerations

The study protocol received approval from the Institutional Review Board (IRB) of the University of Medicine and Pharmacy in Ho Chi Minh City prior to participant recruitment. The study was classified as minimal risk research involving healthcare professionals as expert consultants rather than human subjects. Participation was entirely voluntary, with informed electronic consent obtained from all participants before accessing the first questionnaire.

Participant anonymity was preserved throughout the study through several mechanisms:

- Individual responses were never shared with other participants.
- Qualitative comments were anonymized before inclusion in group feedback.
- The final report presented aggregated results without attributing responses to individual participants.

### 2.7. Statistical Software and Reporting

All statistical analyses were conducted using Epidata and MS Excel for chart drawing. Results are reported according to established guidelines for Delphi studies in healthcare research.

## 3. Results

### 3.1. Expert Panel Characteristics

A total of 35 invitations were sent to potential participants, with 26 experts responding to the first round, yielding an initial response rate of 74.3%. The response rate for the second round was as follows: 21 of 26 Round 1 participants completed Round 2 (80.8% retention rate).

#### 3.1.1. Specialty Distribution

Most participants (69.2%, n = 18) specialized in pulmonology, followed by general internal medicine (15.4%, n = 4), tuberculosis and lung diseases (11.5%, n = 3), and clinical allergy and immunology (3.8%, n = 1).

#### 3.1.2. Institutional Affiliation

Most participants worked in the public sector (76.9%, n = 20), 19.2% (n = 5) worked in the private sector, and 3.8% (n = 1) worked in both public and private sectors.

#### 3.1.3. Level of Care

The panel represented various levels of healthcare delivery: 53.8% (n = 14) worked at the central level, 42.3% (n = 11) at the provincial/city level, and 3.8% (n = 1) at the district level.

#### 3.1.4. Geographic Distribution

Participants represented diverse geographic regions across Vietnam: Mekong River Delta (34.6%, n = 9), Southeast Region (26.9%, n = 7), Red River Delta (23.1%, n = 6), North Central Region (7.7%, n = 2), South Central Coast (3.8%, n = 1), and Northern Midlands and Mountains (3.8%, n = 1).

#### 3.1.5. Clinical Experience

Participants demonstrated substantial clinical expertise with the mean years of experience in respiratory and pulmonary diseases of 11.04 ± 6.76 years (range: 5–25 years). Their mean experience in asthma patient care was 10.81 ± 6.68 years (range: 5–25 years). On average, participants treated 22.31 ± 21.91 asthma patients per week (range: 5–80 patients).

The means, medians, and percentage agreement for each item in both rounds are presented in Table 1 and Table 2. Detailed round-by-round results, including rating percentages, are provided in the Supplementary Materials.

### 3.2. Round 1 Resutls

#### 3.2.1. Perceptions of Scientific Consensus and Number of Zones of Asthma Action Plan

The panel did not reach an agreement that “there is still no scientific consensus to support the components and interventions of an asthma action plan”. Of the participants, 42.3% (n = 11) suggested a 4-zone APP, 38.5% (n = 10) suggested a 3-zone AAP, and 19.2% (n = 5) suggested a 2-zone APP.

#### 3.2.2. Symptoms, Tools, and Interventions to Be Included in the Action Plan

The panel reached an agreement for all seven suggested symptoms for inclusion in action plans, including *night waking*, *increased reliever use*, *shortness of breath*, *limitation in activity*, *wheezing*, *cough*, and *chest tightness*.

Regarding the inclusion of essential statements, interventions, or tools, the panel reached an agreement for five out of the seven suggested items, including the statements that *asthma requires long-term treatment* and that *asthma is a chronic disease*, as well as that the *instructions to seek emergency care or physician’s visit* and *instructions to change medication* and the *Asthma Control Test (ACT)* should be included in the AAP. An agreement was not reached for *instructions to use OCS* and *PEF measurement*.

#### 3.2.3. Severity Categorization

The definitions of symptoms for each zone achieved agreement for all proposed severity levels. Asthma with no symptoms or symptoms present but not interfering with daily activity might be classified as being in the green zone; asthma with symptoms causing discomfort or mild limitations in usual activity might be upscaled to the yellow zone; and symptoms causing severe limitation in usual activity or night waking might be classified as being in the red zone. Patients with difficulty speaking, sleeping, or walking, dyspnea at rest, or unresolved symptoms with reliever should seek emergency support.

For the signs and symptoms criteria for the green zone, agreement was reached for four out of the five proposed items, including (1) *daytime symptoms occurring fewer than two days per week*, (2) *no night waking due to asthma*, (3) *no activity limitation*, and (4) *PEF ≥ 80% with variability <20%*. The item *Reliever use < 2 times/week* did not achieve agreement.

For the signs and symptoms criteria for the yellow zone, agreement was reached for all six proposed items, including (1) *daytime asthma symptoms occurring 2–5 days per week*, (2) *daytime symptoms causing discomfort*, (3) *reliever use more than twice per week*, (4) *night waking due to asthma 1–3 times per month*, (5) *mild activity limitation due to asthma*, and (6) *PEF >80% with variability of 20–30%*.

For the signs and symptoms criteria for the red zone, agreement was achieved for all six proposed items, including (1) *daytime asthma symptoms 6–7 days per week*, (2) *daytime symptoms causing discomfort*, (3) *reliever use 6–7 times per week*, (4) *night waking due to asthma more than once per week*, (5) *moderate-to-severe activity limitation due to asthma*, and (6) *PEF 60–80% or variability >30%*.

For danger signs requiring emergency care, eight of nine proposed items achieved agreement, including (1) *reliever needed every 2–3 h*, (2) *blue or pale lips*, (3) *shortness of breath at rest*, (4) *inability to speak full sentences*, (5) *symptoms worsening very quickly*, (6) *persistent symptoms despite reliever use*, (7) *waking frequently at night*, and (8) *requiring >12 puffs/day of ICS + Formoterol in the MART (Maintenance And Reliever Therapy) plan*. The items *PEF <60% or variability >30%* did not achieve agreement.

An agreement was achieved for all proposed emergency actions to be performed by patients when danger signs are present, including (1) *calling emergency ambulance services*, (2) *informing on asthma attacks*, (3) *continuing to use reliever while waiting*, (4) *staying calm*, (5) *using up to six extra puffs of reliever if symptoms persist*, and (6) *starting oral corticosteroids if pre-prescribed*.

#### 3.2.4. Suggested Actions for Each Zone or Symptom Severity

Suggested actions for each zone were chosen by the regimen treatment MART (Formoterol + ICS combination) or ICS/LABA + SABA. Details of the agreement for items on suggested actions for each zone or symptom severity is described in Table 2.

For patients on MART, agreement was reached for four of the eight proposed actions, including (1) *no change in asthma maintenance treatment and the use of Formoterol + ICS inhaler as a reliever for green zone*, (2) *patients in the yellow zone should visit a physician if using up to 12 puffs/day of Formoterol + ICS*, (3) *patients in the red zone should seek emergency care or visit a physician if symptoms persist or worsen*, and (4) *the use of Formoterol + ICS as a reliever, up to 12 puffs/day* for all zones. Three items related to *adding oral corticosteroids in the red/yellow zones* did not meet agreement.

For patients on ICS/LABA + SABA, agreement was reached for only two of the nine proposed actions, including (1) *adding ICS to increase ICS dosage by four times or to maximum dosage for 1–2 weeks* for *yellow zone* and (2) *seeking emergency care or visiting a physician if symptoms persist or worsen* for the *yellow/red zones*. Items involving continuing regular controller for the green zone or the increase in ICS to the maximum dose with frequent SABA and the addition of oral corticosteroids in the red or yellow zones did not reach agreement.

### 3.3. Round 2 Resutls

#### 3.3.1. Perceptions of Scientific Consensus

In Round 2, agreement was reached for the statement that there is still no scientific consensus to support the components (95.2%) and interventions (80.9%) of an asthma action plan.

#### 3.3.2. Symptoms, Tools, and Interventions to Include in AAP

Neither of the two re-evaluated items, (1) *instructions to use oral corticosteroids* and (2) *home PEF measurement* reached agreement.

##### Severity Categorization

The item *reliever use fewer than two times per week* for the green zone achieved 90.5% agreement. Additionally, *PEF < 60% or PEF change > 30%* as danger signs did not achieve agreement.

#### 3.3.3. Suggested Actions for Each Zone or Symptom Severity

Patient Actions for MART Regimen: none of the four evaluated items achieved agreement (*No change in asthma maintenance treatment*) and the other three items involving oral corticosteroid use).

Patient Actions for ICS/LABA + SABA Regimen: only two of the seven items achieved agreement, including (1) *continue regular controller (ICS/LABA) plus SABA as needed* achieved for the green zone and (2) *add ICS to increase dosage by four times or to maximum for 1–2 weeks* for the red zone. Both “*increase dosage of ICS plus add frequent SABA*” *for* the yellow and red zone and all items involving oral corticosteroid use in red and yellow zones remained below the agreement threshold.

### 3.4. Summary of Round 1 vs. Round 2

Across the two Delphi rounds, several areas demonstrated stable agreement, particularly for symptom-based criteria in the green, yellow, and red zones, most proposed danger signs, the core components of asthma action plans, and the recommended emergency actions.

In Round 2, participants reached an agreement for *reliever use fewer than two times per week* as a green zone criterion, and agreement was also established for *stepping up inhaled corticosteroids in the red zone* for patients on ICS/LABA plus SABA.

Despite these improvements, some items continued to lack agreement. These included reliance on PEF thresholds in both the yellow and red zones as well as for danger signs, the timing and dosing of oral corticosteroids in both MART and ICS/LABA + SABA regimens, and the role of home PEF monitoring. Items not achieving agreement are described in Table 3.

### 3.5. Qualitative Feedback

Panelist feedback provided valuable insights into action plan optimization:

1. PEF Monitoring Concerns
  Multiple participants expressed concerns about PEF monitoring feasibility: “The use of PEF in asthma monitoring is not yet suitable for practical management in Vietnam. The focus should be on clinical signs that are easy for patients to recognize and assess.” Another participant recommended: “Since most asthma patients in Vietnam do not use PEF monitoring at home, this tool should be considered for removal from the asthma action plan in Vietnam to improve its feasibility.”
2. Oral Corticosteroid Administration
  Several participants opposed patient self-administration of oral corticosteroids: “Patients should not be instructed to self-administer oral corticosteroids” was mentioned by multiple participants across both rounds.
3. Emergency Care Recommendations
  Participants suggested immediate emergency care protocols: “Seek emergency medical care in the red zone regardless of response to reliever medication; seek emergency care in the yellow zone if adding ICS does not improve symptoms or if symptoms worsen.”
4. Additional Emergency Instructions
  Participants recommended comprehensive emergency protocols including the self-monitoring of SpO_2_ if equipment is available. Immediate oxygen administration at 5 L/min if available. Nebulized reliever medication with specific drug and dosage. Ensure accompaniment for patient support. Positioning and breathing techniques: “Sit upright, stay calm, breathe slowly, use a fan gently”
5. Structural Recommendations
  Some participants favored simplification: “Divide into 2 zones: Zone 1—stable when no warning signs are present; Zone 2—if any single warning sign appears.” Others emphasized practical clinical signs over objective measurements for patient self-assessment.
6. Treatment Protocols
  For severe exacerbations, panelists recommended: “In patients already using ICS/LABA or ICS/SABA, entering the red zone should prompt immediate addition of systemic corticosteroids for 5–7 days, combined with increasing ICS to the maximum dose for 1–2 weeks, and using SABA regularly every 4–6 h.”

## 4. Discussion

### 4.1. Principal Findings

This Delphi study successfully established expert agreement among Vietnamese physicians on key structural and content elements of AAPs; however, it also revealed some areas of disagreement. The high response rate and diverse geographic representation across Vietnam’s healthcare regions demonstrate strong engagement from the respiratory medicine community. The panel’s substantial clinical experience and significant patient volume ensure that agreement recommendations reflect real-world clinical expertise and practical implementation considerations.

The study achieved agreement on fundamental symptom-based criteria for action plan zones, core symptoms and actions for emergencies and zones, and essential educational components, but failed to reach agreement on peak expiratory flow (PEF) monitoring integration and patient self-administration of oral corticosteroids (OCSs). These findings provide valuable insights into the contextual factors that influence AAP design and implementation.

GINA recommends that a comprehensive written asthma action plan should encompass (1) an elucidation of the indicators that enable the parent or caregiver to identify the deterioration of symptom management, (2) the pharmacological interventions that should be administered, (3) the timing and manner of securing medical assistance, which includes contact information for available emergency services (e.g., physicians’ offices, emergency departments, hospitals, ambulance services, and emergency pharmacies) [5]. This consensus results are aligned with GINA. The AAP ought to incorporate several essential statements of asthma, indicators for self-assessment, a standardized asthma control questionnaire, protocols for adjustments in maintenance therapy, and guidelines for emergency circumstances. The classification of AAP zones should be predicated on the frequency and intensity of symptoms. The actions taken by patients should be customized according to their prescribed therapeutic regimen (MART or ICS/LABA + SABA).

The experts favored a 4-zone and 3-zone system over a 2-zone system, an approach that aligns with international trends toward more granular symptom monitoring and graduated response protocols [1,11,12]. The AAPs from the National Asthma Council of Australia have a 4-zone system [11] while Canadian AAPs have a 3-zone system [12]. In the United Kingdom, there are varieties of AAPs, but they also have a 4-zone or 3-zone system [13]. The unanimous or near-unanimous agreement on core symptoms and emergency protocols reflects alignment with international guidelines and evidence-based practice [5,27].

The panel agreed that AAPs may include indicators for self-assessment, a standardized asthma control test, and protocols for medication dose adjustment without the need for monitoring devices. Such an approach may be suitable for countries with limited resources, although peak flow monitoring has been shown to improve asthma outcomes. [1].

### 4.2. Non-Agreement Items

#### 4.2.1. Oral Corticosteroid Self-Administration: Safety and Stewardship Concerns

The lack of agreement on oral corticosteroid protocols across both MART and ICS/LABA + SABA regimens in this consensus indicates systematic expert concerns about patient self-administration of systemic corticosteroids and legitimate safety issues. Evidence showing that action plans including oral corticosteroid instructions can improve outcomes when properly implemented [1]; however, the inclusion of oral corticosteroid instructions in action plans [1] must be balanced against growing recognition of cumulative corticosteroid-related adverse effects and the need for careful patient selection and monitoring [28]. Recent Delphi studies on OCS tapering have highlighted the complexity of corticosteroid management and the need for structured protocols and specialist oversight [29]. The Vietnamese consensus outcome reflects these safety priorities and may represent a more cautious approach to the self-management of exacerbation. Multiple experts explicitly opposed patient self-administration.

In Vietnam, oral corticosteroids are easily accessible without a prescription, raising substantial concerns regarding potential misuse, safety, and the need for appropriate monitoring and medical supervision during therapy. Based on the authors’ clinical experience, many Vietnamese patients with asthma—particularly those without regular maintenance treatment—tend to use oral corticosteroids during episodes of severe symptoms or exacerbations.

The lack of agreement on OCS administration raised a debate about the balance between patient empowerment and medication safety. While some guidelines support patient-initiated OCS for exacerbations, implementation requires robust patient education, clear protocols, and reliable follow-up systems that may be challenging to establish consistently across diverse healthcare settings [30].

The use of oral corticosteroids for the treatment of asthma was also common in primary care physicians in Vietnam [10]. The panel expressed concerns that both patients and practitioners might misuse systemic corticosteroids. The panel’s consistent disagreement on self-administration indicates that there exists a necessity to regulate the inappropriate usage of OCS.

#### 4.2.2. PEF Monitoring

The implementation of PEF monitoring is another challenge with significant variation in PEF utilization in routine clinical practice; many healthcare providers omit PEF instructions from action plans due to concerns about patient technique, device availability, and interpretation challenges [31]. In addition, PEF devices could not be found in Vietnam in most circumstances. It can increase the cost of care and put the burden on patients for their asthma adherence.

The increase in ICS dosage and addition of SABA for patients treated with the ICS/LABA + SABA regimen.

The panel agreed on quadrupling ICS doses for 1–2 weeks for patients on ICS/LABA + SABA in both the yellow zone and red zone but failed to achieve agreement for increasing the dose of ICS and adding frequent SABA (2 puffs q6–8 h) use on those zones.

Large pragmatic trials have demonstrated that patient-implemented self-management plans instructing temporary quadrupling of ICS dose can reduce the time to first severe exacerbation and overall exacerbation risk, with cost-effectiveness analyses supporting this approach [32]. The FAST trial, involving over 1900 patients, showed that quadrupling ICS dose at the first sign of asthma deterioration reduced severe exacerbations by 19% compared to usual care [32]. However, this positive evidence is countered by systematic reviews of blinded randomized controlled trials. A recent Cochrane review found that increasing ICS at the first sign of exacerbation probably does not reduce the odds of needing rescue oral corticosteroids compared with stable dosing (OR 0.97, 95% CI 0.76–1.25) in adults and children with mild-to-moderate asthma [33]. The panel reached agreement about ICS step-up only at round 2, which might show their concerns on the efficacy of ICS step-up.

The inclusion of frequent SABA use (every 4–8 h) in the proposed items likely contributed significantly to expert concerns. Extensive observational evidence demonstrates that SABA overuse is consistently associated with worse asthma outcomes. Large real-world datasets show that prescribing or using ≥3SABA canisters per year is associated with higher rates of severe exacerbations and can identify patients at elevated risk [34]. Multiple international cohort studies have linked SABA over-prescription to increased exacerbations, hospital admissions, and in some populations, higher mortality, with clear dose-dependent relationships [35,36]. The proposed combination of high-dose ICS with frequent scheduled SABA use creates a particularly concerning scenario from a safety perspective.

### 4.3. Methodological Strengths and Limitations

#### 4.3.1. Study Strengths

This study employed a rigorous Delphi methodology with appropriate consensus thresholds and achieved excellent expert participation and retention rates. The geographic diversity of experts across Vietnam’s healthcare regions and the substantial clinical experience of participants enhance the generalizability of findings within the Vietnamese healthcare context. The inclusion of both quantitative consensus measurement and qualitative feedback provided rich insights into expert reasoning and implementation concerns.

The two-round design allowed for expert reflection and opinion refinement, with several items achieving agreement in Round 2 that had failed to meet thresholds initially. This iterative process strengthens the validity of final agreement recommendations and provides insight into areas of persistent disagreement that require alternative approaches.

#### 4.3.2. Limitations and Considerations

The study focused exclusively on Vietnamese healthcare providers, limiting direct generalizability to other healthcare systems and cultural contexts. However, this focused approach enables the development of contextually appropriate recommendations while contributing to the global understanding of factors influencing asthma action plan design and implementation.

The predominance of pulmonologists in the panel may have influenced perspectives on complex treatment decisions and emergency management protocols. While this expertise enhances clinical validity, a broader inclusion of primary care providers might have provided additional insights into implementation feasibility in routine practice settings.

The study did not include patient perspectives on action plan preferences and usability, which represent important considerations for successful implementation. Future research should incorporate patient and caregiver input to ensure that expert consensus recommendations align with end-user needs and capabilities.

### 4.4. Future Research Directions

The consensus recommendations provide a foundation for developing standardized asthma action plans that can be evaluated through implementation research and clinical effectiveness studies. Future research should examine patient outcomes, healthcare utilization, and quality of life impacts associated with consensus-based action plans compared to current practice variations.

## 5. Conclusions

The asthma action plan should incorporate essential statements pertaining to asthma, criteria for the self-monitoring of symptoms, an objective asthma control questionnaire, directives for modifications of maintenance pharmacotherapy, and guidelines for emergency situations. The classification of AAP zones should be based on the frequency and intensity of symptoms. Patient actions ought to be customized according to their specific treatment protocols and dosing adjustment based on symptom recognition. The AAP may not encompass the monitoring of peak expiratory flow rates or the self-administration of oral corticosteroids within both the MART and ICS/LABA + SABA therapeutic regimens, and it may not recommend the concurrent use of SABA with ICS dose escalation in the ICS/LABA + SABA treatment regimen.

This consensus may serve as a foundation for the future development of a tailored AAP for Vietnam, as it reflects the views of Vietnamese experts.

## Figures and Tables

**Table 1:** Agreement of items on content and severity categorization of asthma action plan for two rounds of survey.

Item	Mean ± SD	Median	Percent of Agreement *
**Round 1**			
**Signs or symptoms which should be included in the asthma action plan**			
Cough	4.19 ± 0.801	4.00	84.6
Wheezing	4.46 ± 0.582	4.50	96.2
Chest tightness	4.19 ± 0.801	4.00	84.6
Shortness of breath	4.5 ± 0.583	5.00	96.2
Night waking due to symptoms	4.46 ± 0.508	4.00	100
Increased reliever use	4.5 ± 0.51	4.50	100
Limitation in activity	4.35 ± 0.562	4.00	96.2
**Information, tools, or interventions that should be included in the asthma action plan**			
Asthma is a chronic disease	4.31 ± 0.618	4.00	92.3
Asthma requires long-term treatment	4.46 ± 0.582	4.50	96.2
Home peak flow measurement **	3.69 ± 1.05	4.00	61.60
The Asthma Control Test (ACT)	4.04 ± 0.774	4.00	80.8
Instructions to change medication	4.19 ± 0.634	4.00	88.5
Instructions to seek emergency care or physician’s visit	4.54 ± 0.508	5.00	100
Instructions to use oral corticosteroids **	3.73 ± 0.962	4.00	65.40
**The definitions of symptoms for each zone**			
Green Zone: No symptoms or symptoms present but not interfering with daily activity	4.12 ± 0.952	4.00	88.46
Yellow Zone: Symptoms causing discomfort or mild limitation in usual activity	4.19 ± 0.849	4.00	92.31
Red Zone: Symptoms causing severe limitation in usual activity or night waking	4.15 ± 0.834	4.00	92.31
Very severe/Need for emergency care: Difficulty speaking, sleeping, or walking; dyspnea at rest; reliever not helping	4.31 ± 0.549	4.00	96.15
**Classification of signs and symptoms for green zone**			
Daytime asthma symptoms < 2 days/week	3.85 ± 0.834	4.00	80.77
Reliever use < 2 times/week **	3.73 ± 1.002	4.00	73.08
No night waking due to asthma	4.31 ± 0.471	4.00	100.00
No activity limitation due to asthma	4.27 ± 0.533	4.00	96.15
PEF ≥ 80% and PEF change < 20%	3.96 ± 0.824	4.00	80.77
**Classification of signs and symptoms for yellow zone**			
Daytime asthma symptoms 2–5 days/week	4.12 ± 0.653	4.00	92.31
Daytime asthma symptoms causing discomfort	4.12 ± 0.653	4.00	92.31
Reliever use > 2 times/week	4.12 ± 0.653	4.00	92.31
Night waking due to asthma 1–3 times/month	4.15 ± 0.464	4.00	96.10
Mild activity limitation due to asthma	4.19 ± 0.634	4.00	96.15
PEF > 80% and PEF change 20–30%	3.92 ± 0.935	4.00	80.77
**Classification of signs and symptoms for red zone**			
Daytime asthma symptoms 6–7 days/week	4.23 ± 0.514	4.00	96.15
Daytime asthma symptoms causing discomfort	4.23 ± 0.514	4.00	96.15
Reliever use 6–7 times/week	4.19 ± 0.491	4.00	96.15
Night waking due to asthma > 1 times/week	4.08 ± 0.628	4.00	92.31
Moderate-to-severe activity limitation due to asthma	4.27 ± 0.533	4.00	96.15
PEF 60–80% or PEF change > 30%	3.96 ± 0.824	4.00	80.77
**Danger signs that require emergency care**			
Symptoms worsen very quickly	4.23 ± 0.71	4.00	84.62
Persistent symptoms despite reliever use	4.19 ± 0.749	4.00	80.80
Reliever needed every 2–3 h	4.31 ± 0.549	4.00	96.15
Waking frequently at night due to asthma	4.15 ± 0.675	4.00	84.62
Shortness of breath at rest	4.35 ± 0.629	4.00	92.31
Unable to speak full sentences	4.35 ± 0.629	4.00	92.31
Blue or pale lips	4.42 ± 0.578	4.00	96.15
PEF < 60% or PEF change > 30% **	4.04 ± 0.916	4.00	76.92
Needing >12 puffs/day of Formoterol + ICS (MART plan)	4.19 ± 0.749	4.00	88.46
**Emergency actions by patients when danger signs are present**			
Call emergency ambulance service	4.31 ± 0.736	4.00	92.31
Inform emergency services	4.31 ± 0.736	4.00	92.31
Continue using reliever	4.42 ± 0.578	4.00	96.20
Sit upright and stay calm	4.19 ± 0.634	4.00	88.46
Use up to 6 extra puffs of reliever if symptoms persist	4.08 ± 0.688	4.00	88.46
Start oral corticosteroids	4.12 ± 0.653	4.00	84.62
**Round 2**			
Home peak flow measurement	3.48 ± 1.47	4.00	57.14
Instructions to use oral corticosteroids	3.14 ± 1.526	4.00	61.90
Reliever use < 2 times/week should be classified as green zone	3.95 ± 0.74	4.00	90.48
PEF < 60% or PEF change > 30% should be classified as red zone	3.1 ± 1.221	3.00	33.33

* Percent of agreement was calculated by the sum of the percentages of participants who selected “Agree” and “Strongly Agree”. ** Items did not reach consensus in round 2.

**Table 2:** Agreement of items on patients actions for each zone of asthma action plan for two rounds of survey.

Item	Round 1			Round 2		
	Mean ± SD	Median	Percent of Agreement *	Mean + SD	Median	Percent of Agreement *
**Patient actions for MART regimen**						
Green Zone: No change in asthma maintenance treatment and the use of Formoterol + ICS inhaler as a reliever	4.04 ± 0.824	4.00	84.62	NA		
Yellow Zone: No change in asthma maintenance treatment and the use of Formoterol + ICS inhaler as a reliever	3.46 ± 0.989	4.00	61.54	3.71 ± 0.717	4.00	76.19
Yellow Zone: Visit a physician if using up to 12 puffs/day of Formoterol + ICS	4.12 ± 0.864	4.00	84.62	NA		
Red Zone: Seek emergency care or visit a physician if symptoms persist or worsen	4.38 ± 0.637	4.00	92.31	NA		
Red Zone: Add oral corticosteroid if PEF or FEV_1_ < 60% of personal best or predicted	3.81 ± 0.849	4.00	69.23	3.48 ± 1.209	4.00	61.90
Yellow and Red Zones: Add oral corticosteroid if there is no response to treatment after 2 days	3.65 ± 0.977	4.00	65.38	3.38 ± 1.359	4.00	61.90
Yellow and Red Zones: Oral corticosteroid dose: prednisone 40–50 mg/day or equivalent for 5–7 days	3.54 ± 0.905	4.00	61.54	3.33 ± 1.317	4.00	61.90
All Zones: Use Formoterol + ICS inhaler as a reliever (1 puff as needed), up to 12 puffs/day	4.23 ± 0.815	4.00	92.31		NA	
**Patient actions for Patients on ICS/LABA + SABA**						
Green zone: Continue regular controller (ICS/LABA) + SABA as needed	4 ± 0.98	4.00	76.92	4.14 ± 0.478	4.00	95.24
Yellow zone: Add ICS to increase ICS dosage by 4 times or to maximum dosage for 1–2 weeks	4 ± 0.748	4.00	80.77	NA		
Yellow Zone: Add ICS to increase ICS dosage by 4 times or to maximum dosage for 1–2 weeks + frequent SABA (2 puffs q6–8 h)	3.96 ± 0.871	4.00	76.92	3.29 ± 1.007	3.00	47.62
Red Zone: Add ICS to increase ICS dosage by 4 times or to maximum dosage for 1–2 weeks	3.85 ± 0.925	4.00	73.08	3.71 ± 0.902	4.00	80.95
Red Zone: Add ICS to increase ICS dosage by 4 times or to maximum dosage for 1–2 weeks + frequent SABA (2 puffs q4–6 h)	3.88 ± 0.909	4.00	69.23	3.33 ± 1.155	4.00	57.14
Red Zone: Add oral corticosteroid if PEF or FEV_1_ < 60% of personal best or predicted	3.73 ± 1.041	4.00	65.38	3.33 ± 1.197	4.00	52.40
Yellow and Red Zones: Seek emergency care or visit a physician if symptoms persist or worsen	4.31 ± 0.618	4.00	92.30	NA		
Yellow and Red Zones: Add oral corticosteroids if there is no response to treatment change within 2 days	3.96 ± 0.871	4.00	76.92	3.33 ± 1.317	4.00	57.14
Yellow and Red Zones: Oral corticosteroid dose: prednisone 40–50 mg/day or equivalent for 5–7 days	3.85 ± 0.881	4.00	69.30	3.43 ± 1.287	4.00	61.90

* Percent of agreement was calculated by the sum of the percentage of the participants who selected “Agree” and “Strongly Agree”.

**Table 3:** Items not achieving agreement.

Item	Round 1 Agreement (%)	Round 2 Agreement (%)
**Peak Expiratory Flow (PEF) Monitoring**		
Home PEF measurement should be included in the asthma action plan.	61.6	57.1
PEF < 60% or PEF change > 30% as a danger sign.	76.9	33.3
Initiate oral corticosteroid based on reduction in PEF or change in PEF (for all zone of the asthma action plan)	No agreement for all proposed items in each zone	No agreement for all proposed items in each zone
**Oral Corticosteroids**		
Add oral corticosteroids (for both patients on MART and on ICS/LABA + SABA) in all zones.	No agreement for all proposed items	No agreement for all proposed items
Dosage of oral corticosteroid use (for both patients on MART and on ICS/LABA + SABA) in all zones.	No agreement for all proposed items	No agreement for all proposed items

## Data Availability

The original contributions presented in this study are included in the article/Supplementary Materials Section. Further inquiries can be directed to the corresponding author.

## Supplementary Materials

The following supporting information can be downloaded at https://www.mdpi.com/article/10.3390/jcm14248640/s1, File S1: Detailed round-by-round results.

## Abbreviations

The following abbreviations are used in this manuscript:

AAP	Asthma action plan
PEF	Peak expiratory flow
OCS	Oral corticosteroids
ICS	Inhaled corticosteroid
LABA	Long-acting beta-agonist
SABA	Short-acting beta-agonist
MART	Maintenance And Reliever Therapy
ACT	Asthma Control Test
GINA	Global Initiative for Asthma
