# Supplementary material for: Vietnamese Consensus on the Structure and Content of Asthma Action Plan
Source: J Clin Med. 2025 Dec 5;14(24):8640. doi: 10.3390/jcm14248640 (PMC12733560; doi:10.3390/jcm14248640)

Developing Vietnamese Expert Consensus on the Structure and Content of Asthma Action Plans Using the Delphi Method

Round 1 result

26 experts participated in round 1.

Section A: Overview of an asthma action plan

1. Q1: Would you agree that there is still no scientific consensus to support the components and interventions of an asthma action plan?

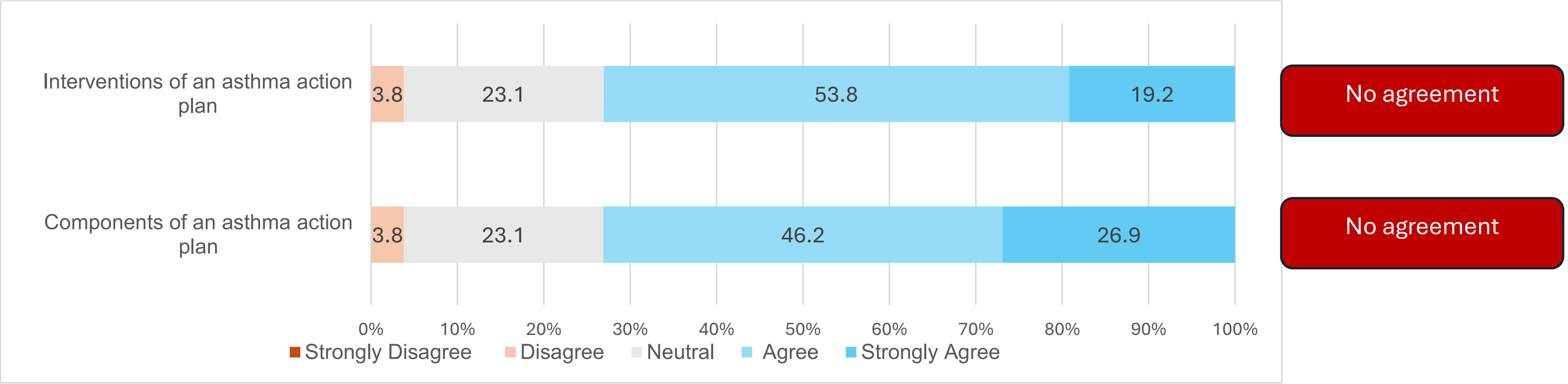

2. Q2: In your opinion, how many zones (distinct sections) should an asthma action plan include to be clear and clinically useful for patients?

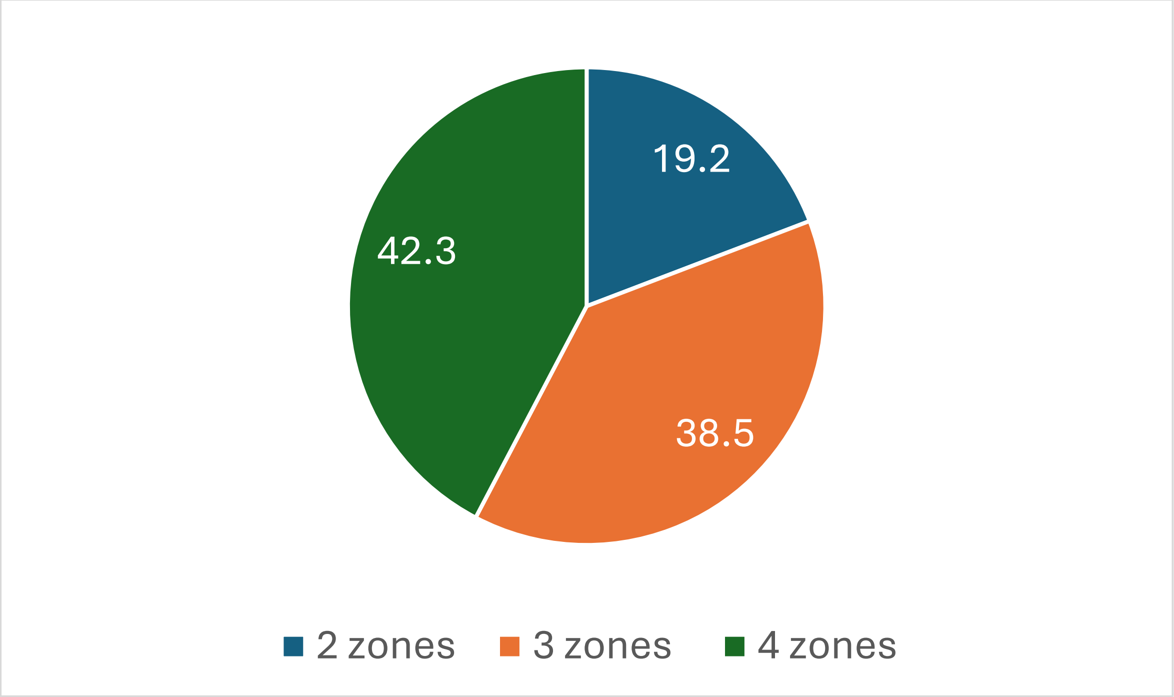

Section B – Symptoms, tools and interventions to Include in the Action Plan

3. Q3: Would you agree that the following signs or symptoms should be included in the asthma action plan?

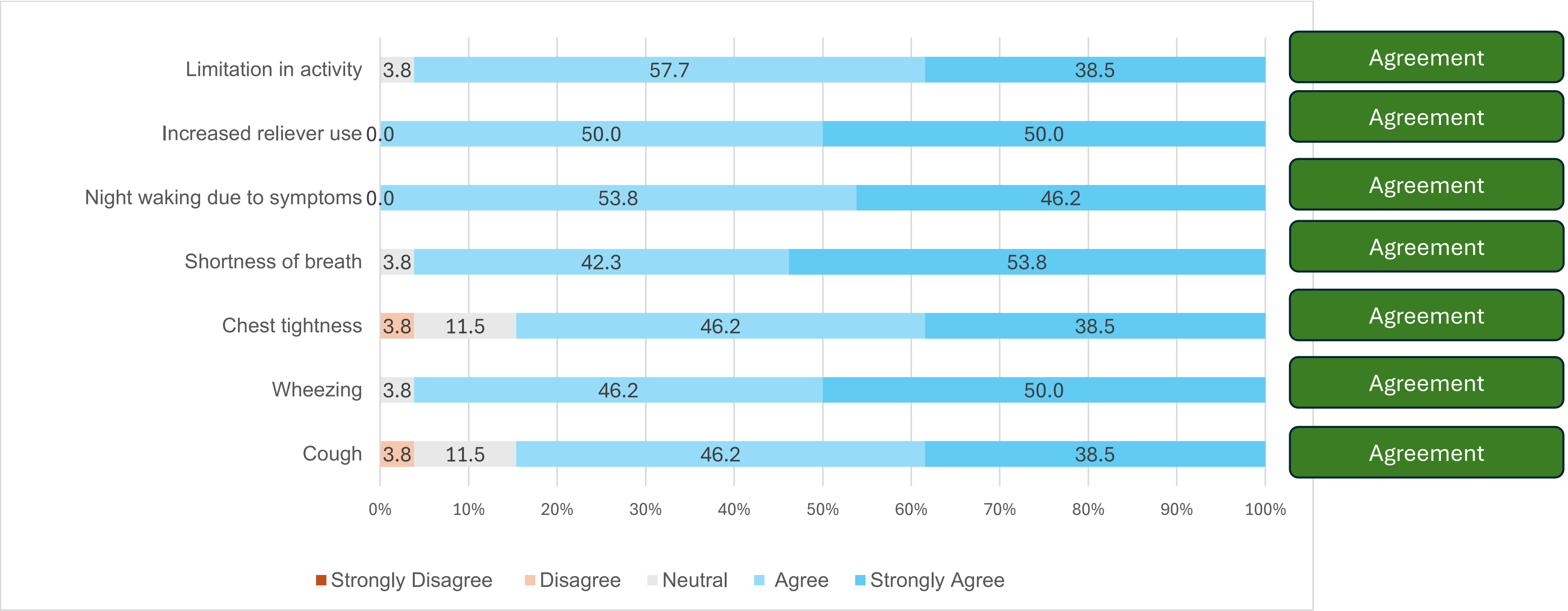

4. Q4: Do you have any suggestions for signs or symptoms that should be included in the asthma action plan?

- Fever, anaphylaxis
- Physical activity limitation

5. Q5: Would you agree that the following information, tools or interventions should be included in the Asthma Action Plan?

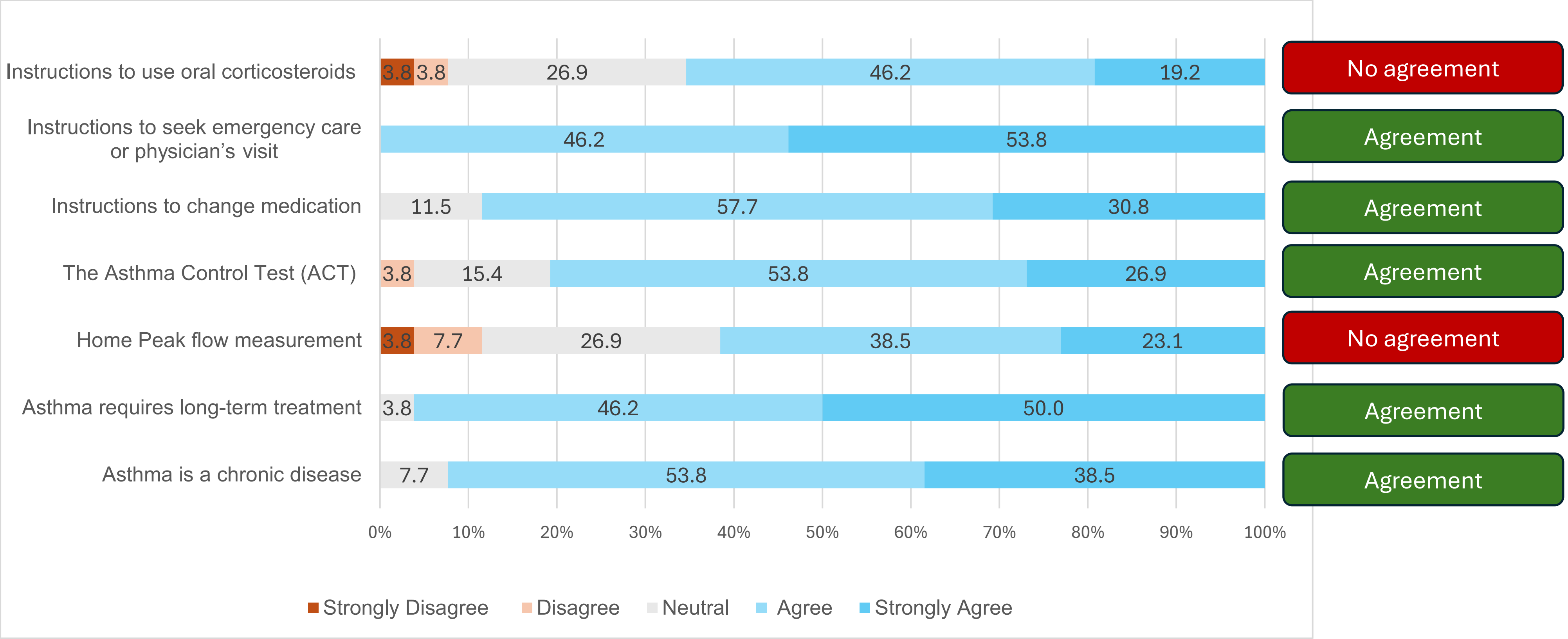

6. Q6: Are there any other symptoms, information, tools or interventions you believe should be included?

- Each unit should have a support hotline
- Provide guidance on initial use of reliever medication at home in case of an acute attack
- Provide instruction on inhaler use

Section C – Severity Categorization

7. Q7: Would you agree that the definitions of symptoms for each zone of an asthma action plan should be classified as following:

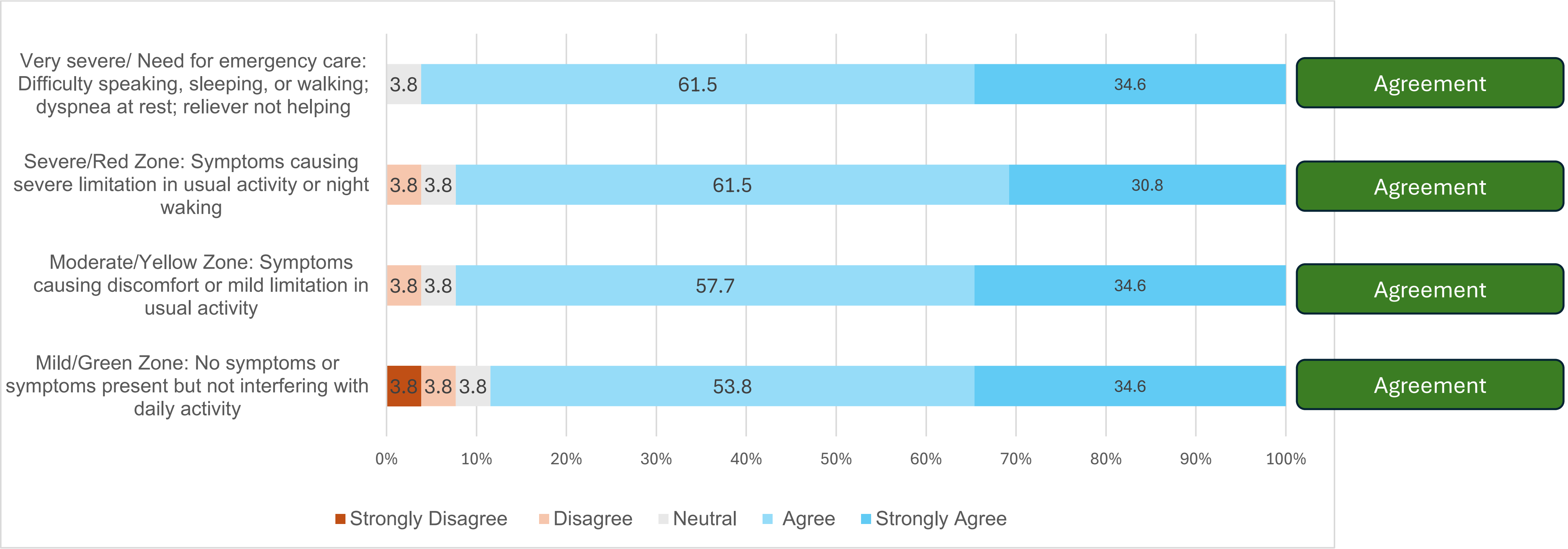

8. Q8: Would you recommend any improvements to these definitions if needed?

- Divide into 2 zones: Zone 1 – stable when no warning signs are present; Zone 2 – if any single warning sign appears
- Chest pain
- Severe/very severe zone requiring emergency care should be classified as the red zone

9. Q9: Would you agree that if a patient has these following signs and symptoms, he or she should be classified in the **green zone** of an asthma action plan?

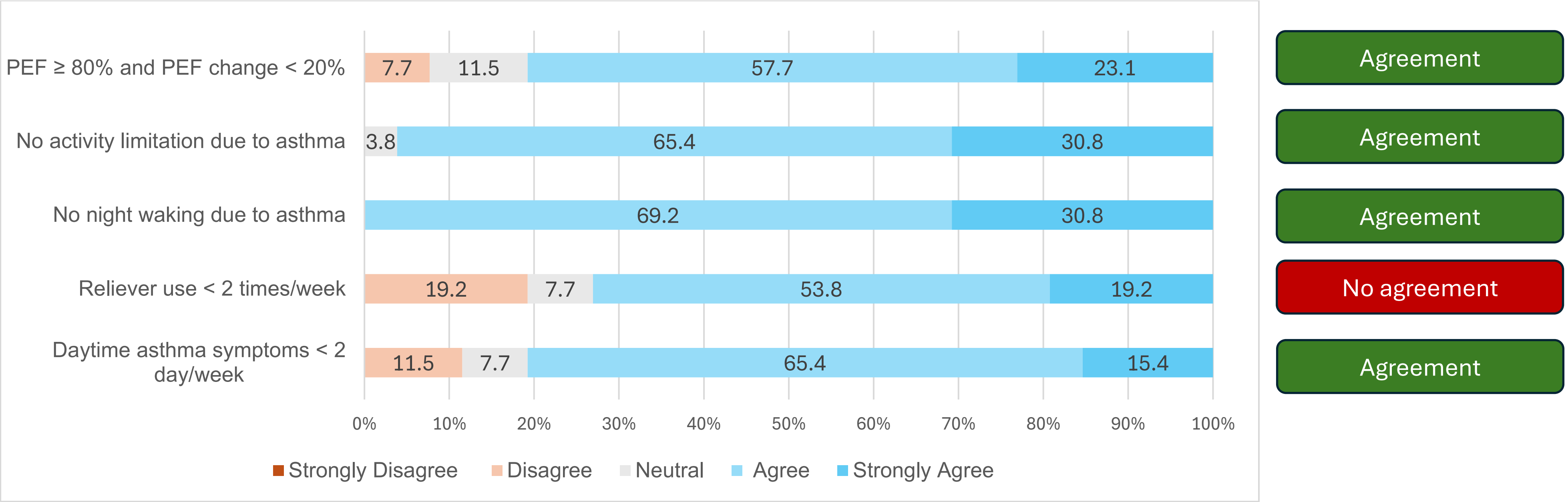

10. Q10: Would you agree that if a patient has these following signs or symptoms, he or she should be classified in the yellow zone of an asthma action plan

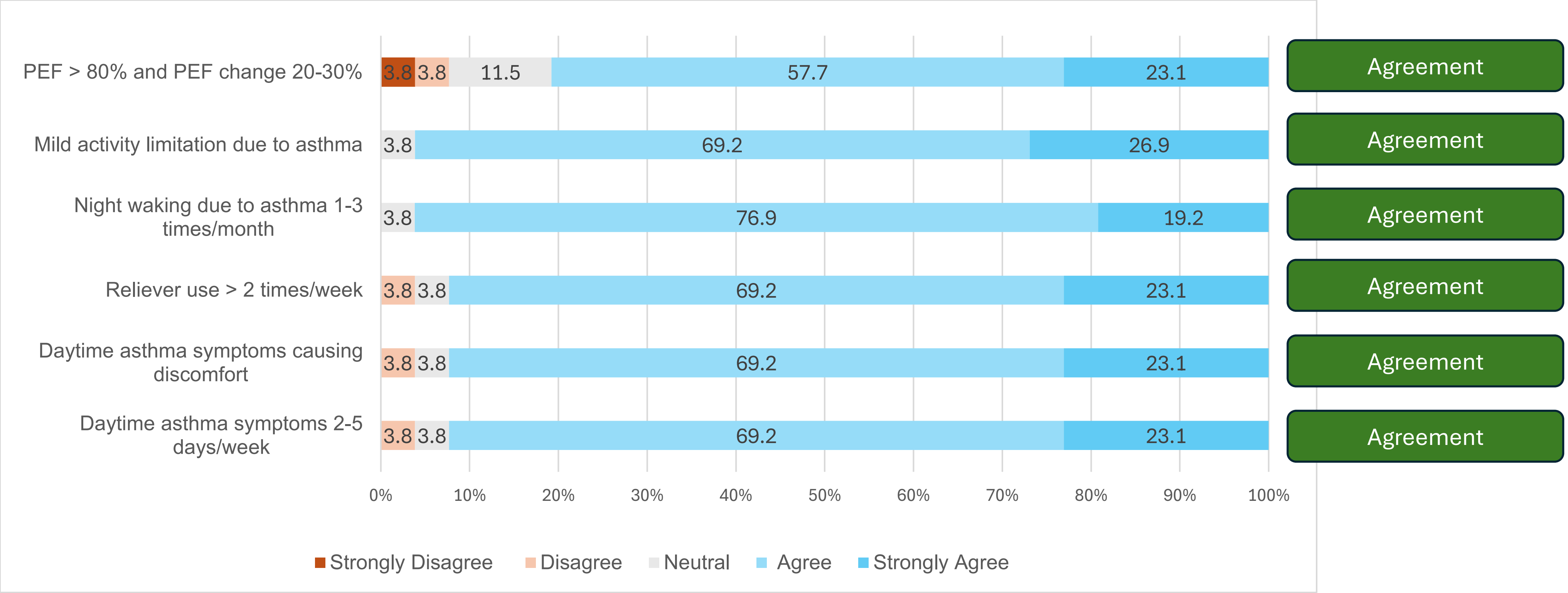

11. Q11: Would you agree that if a patient has these following signs or symptoms, he or she should be classified in the **red zone** of an asthma action plan?

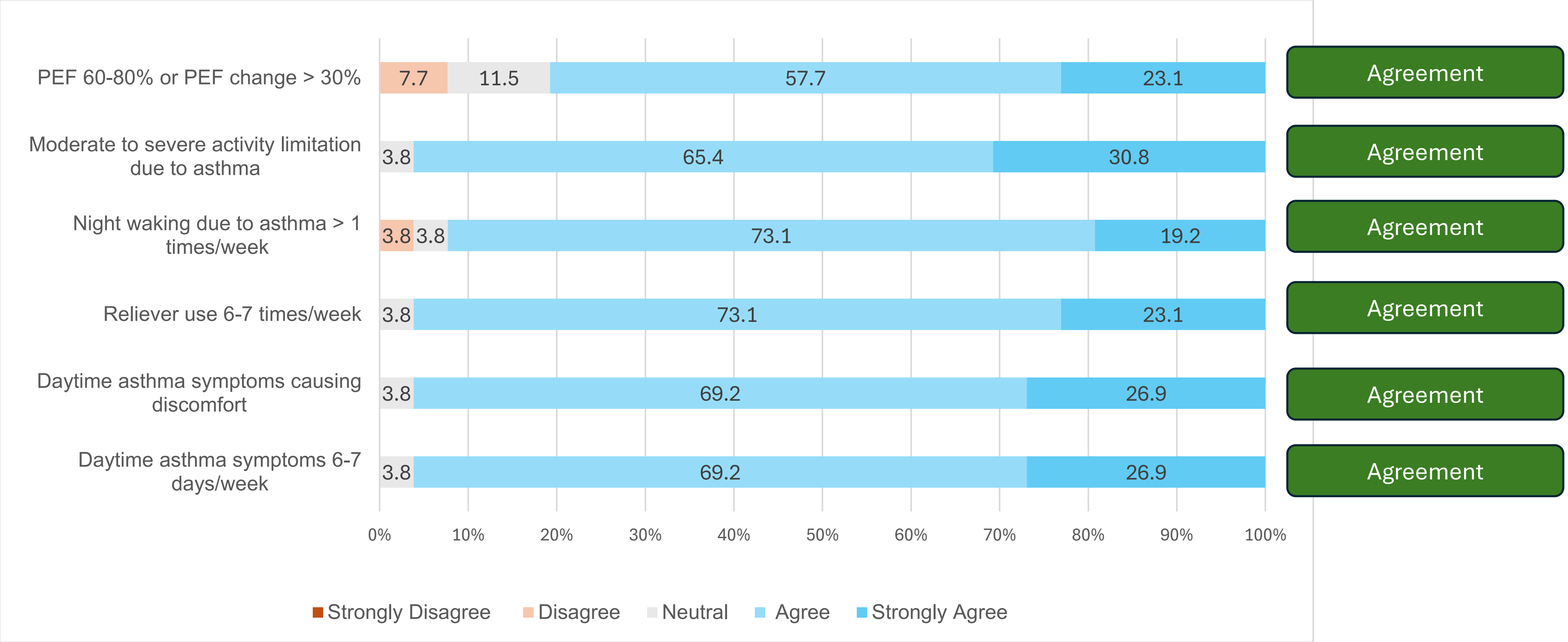

12. Q12: Would you recommend adjusting or adding any signs and symptoms in the zones of an asthma action plan?

- The use of PEF in asthma monitoring is not yet suitable for practical management in Vietnam. The focus should be on clinical signs that are easy for patients to recognize and assess.

13. Q13: Would you agree that each of the following signs or symptoms is as a “danger sign” that indicates the need for emergency care in an asthma action plan?

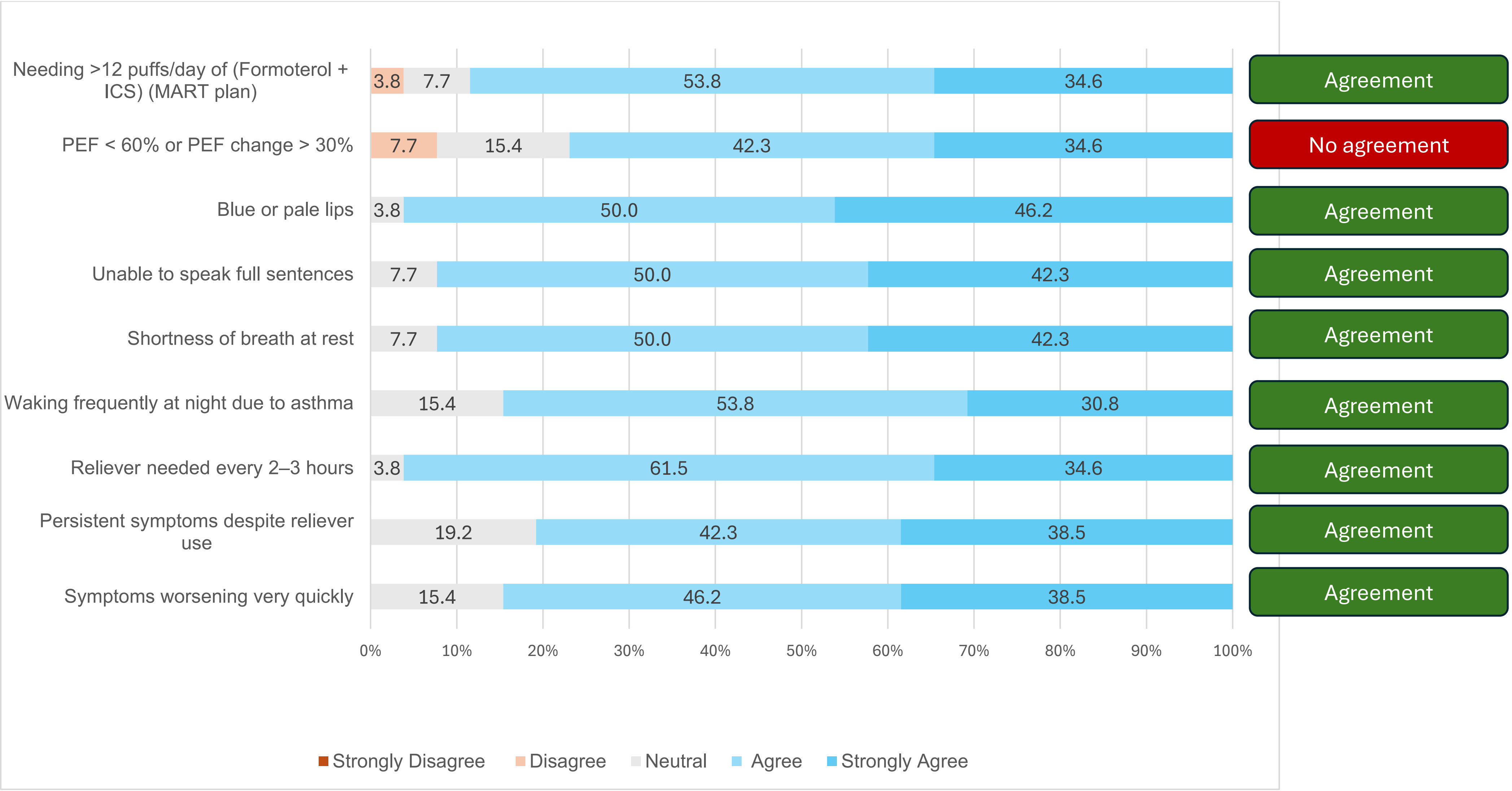

14. Q14: Would you recommend adjusting or adding any danger sign that indicates the need for emergency care to the asthma action plan?

- Used reliever medication two times or more without relief from shortness of breath symptoms

Section D – Recommended Actions for Each Zone/Symptom Severity

15. Q15: Would you agree that the following patient actions should be included in the action plan for Patients on MART (Formoterol + ICS combination) to ensure the appropriateness and feasibility in Vietnam settings?

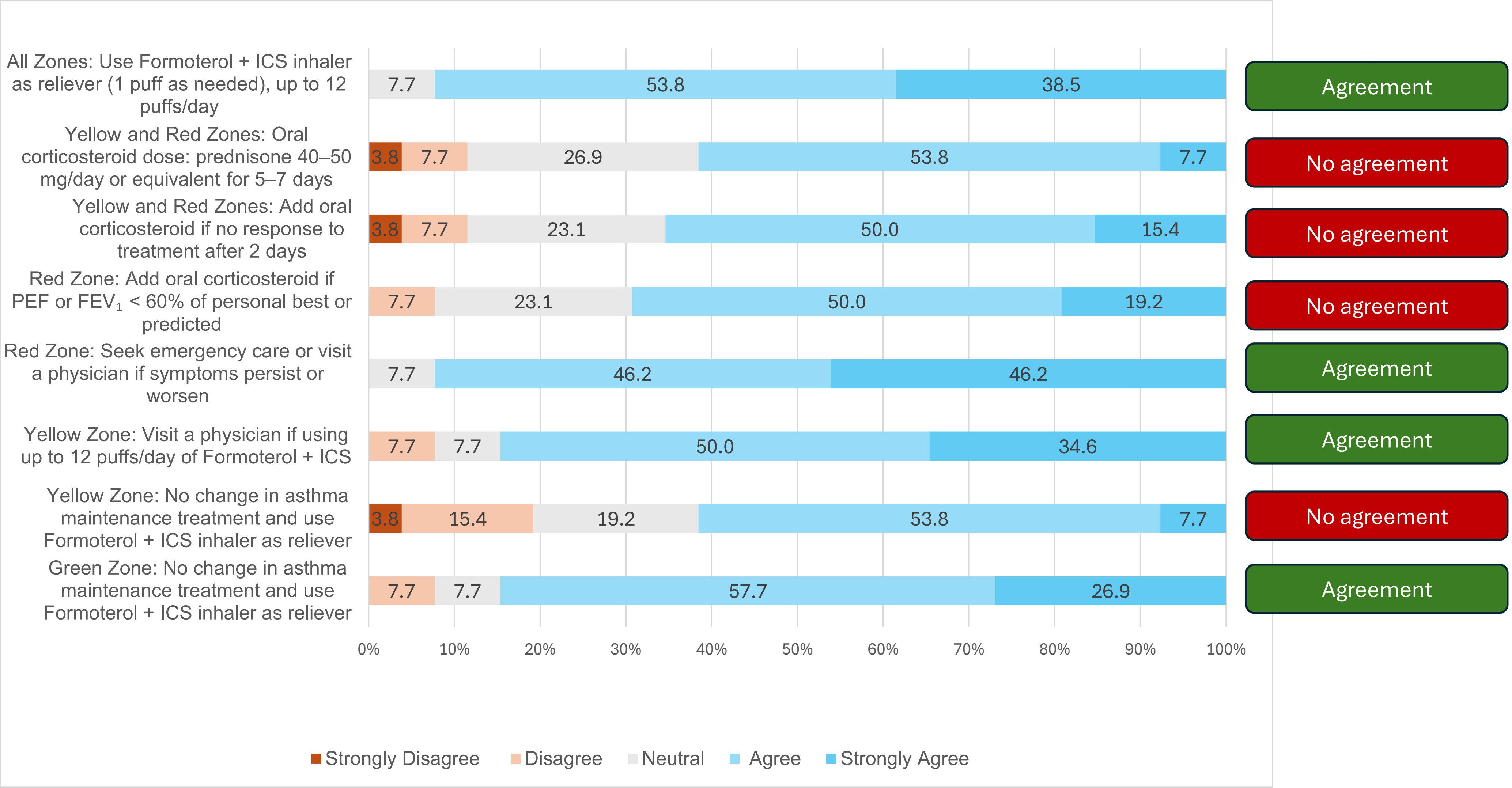

16. Q16: Would you agree that the following patient actions should be included in the action plan for Patients on ICS/LABA + SABA to ensure the appropriateness and feasibility in Vietnam settings?

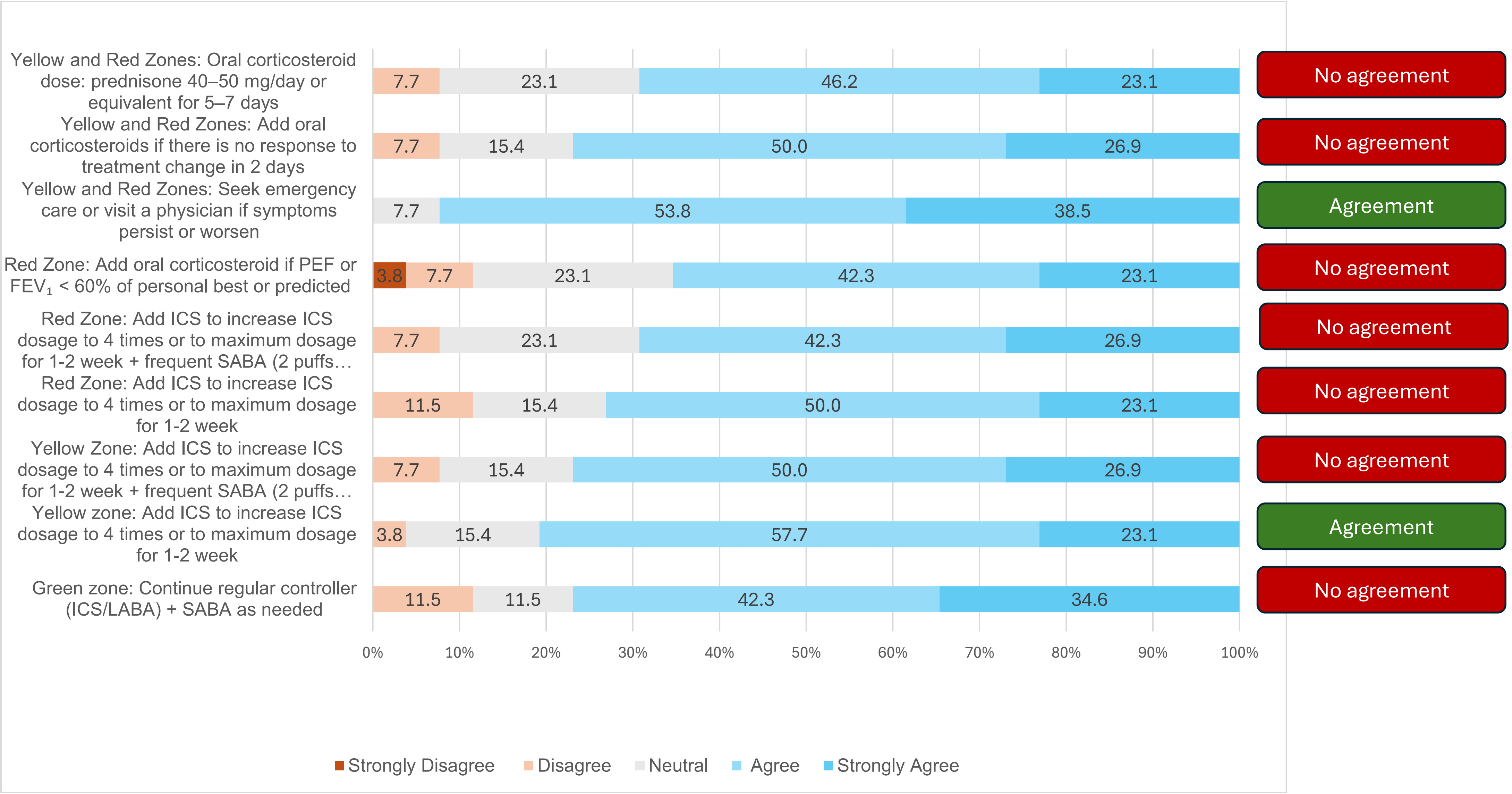

17. Q17: Would you have any additional actions you would recommend including in the asthma action plan? (Applicable to both MART regimen and ICS/LABA + SABA or ICS + SABA regimens)

- Seek emergency medical care in the red zone regardless of response to reliever medication; seek emergency care in the yellow zone if adding ICS does not improve symptoms or if symptoms worsen
- Patients should not be intructed to have self-administer oral corticosteroids

18. Q18: Would you agree that the following emergency actions should be done by patients when danger signs are present?

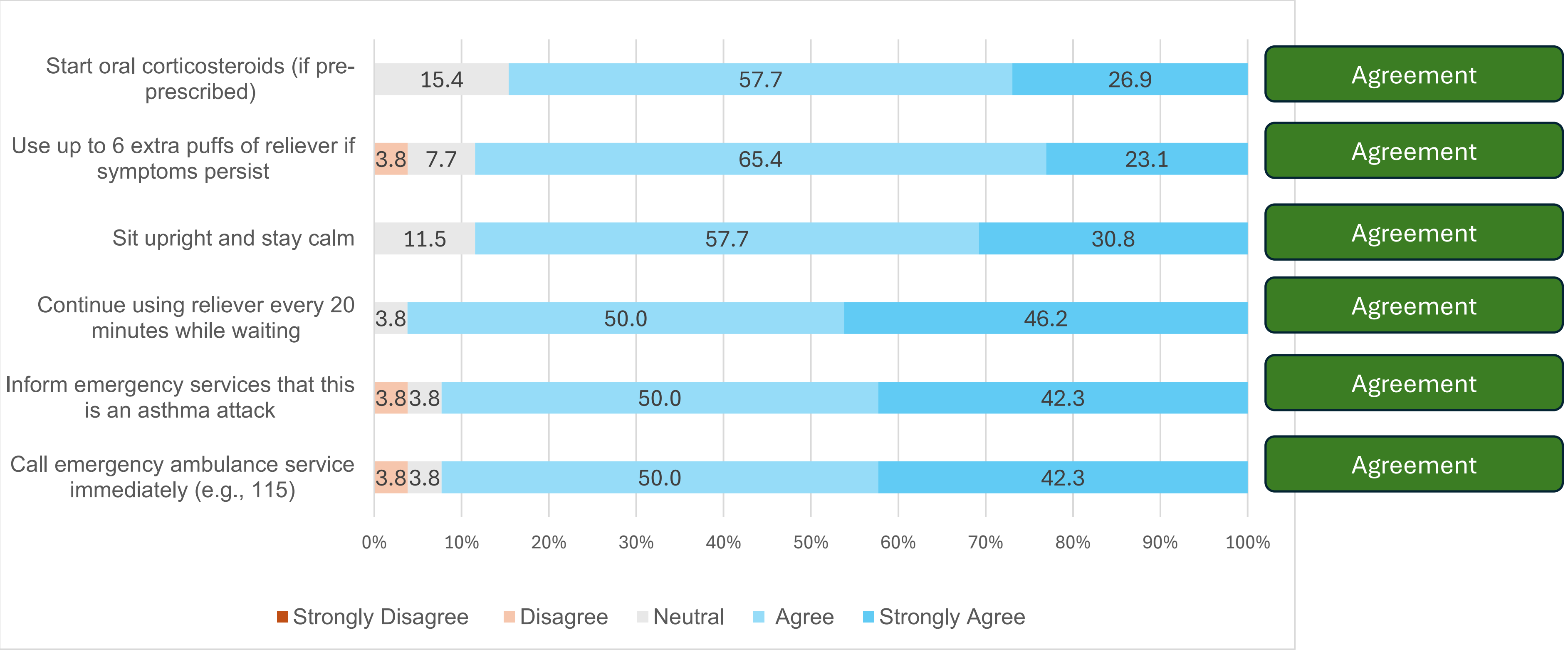

19. Q19: Are there any other symptoms or emergency instructions that should be included in the danger zone of the asthma action plan?
    - Self-monitor SpO_2 if equipment is available
    - Go to the nearest medical facility
    - Cyanosis
    - Sit upright, stay calm, breathe slowly, use a fan gently, use SABA + ICS via nebulizer if available, and call emergency services (115) at the same time
    - Administer oxygen at 5 liters per minute immediately if available
    - Provide reliever medication in nebulized form, with specific drug and dosage
    - Ensure someone accompanies the patient for support

20. Q20: Other recommendations you recommend adding or changing for an asthma action plan?
    N/A

Round 2 result

21 out of 26 experts who participated in round 1 continued to respond in round 2.

Section A: Overview of an asthma action plan

1. Q1: Would you agree that there is still no scientific consensus to support the components and interventions of an asthma action plan?

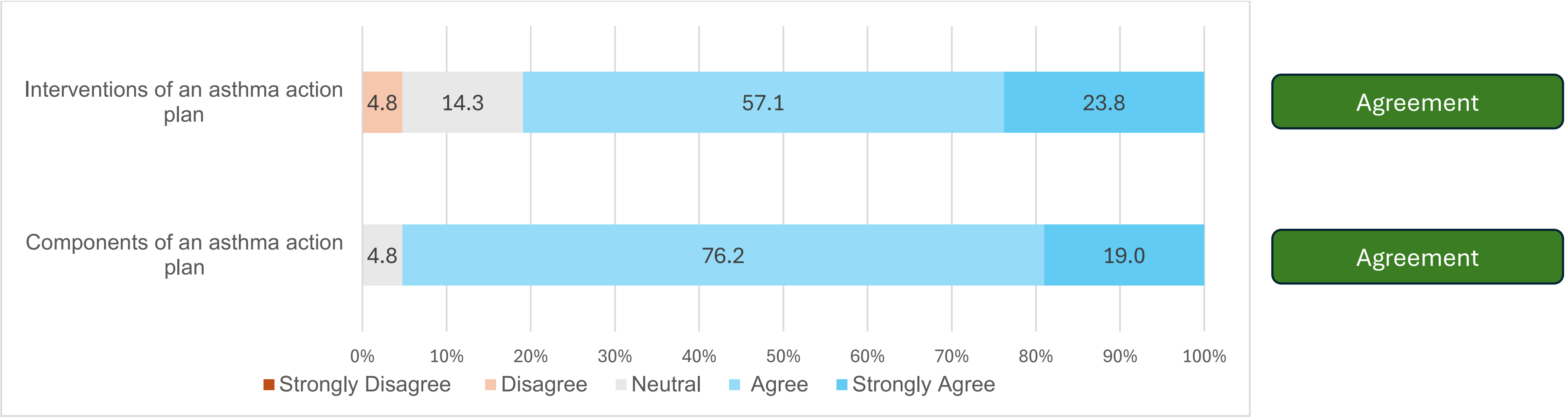

Section B – Symptoms, tools and interventions to Include in the Action Plan

2. Q5: Would you agree that the following information, tools or interventions should be included in the Asthma Action Plan?

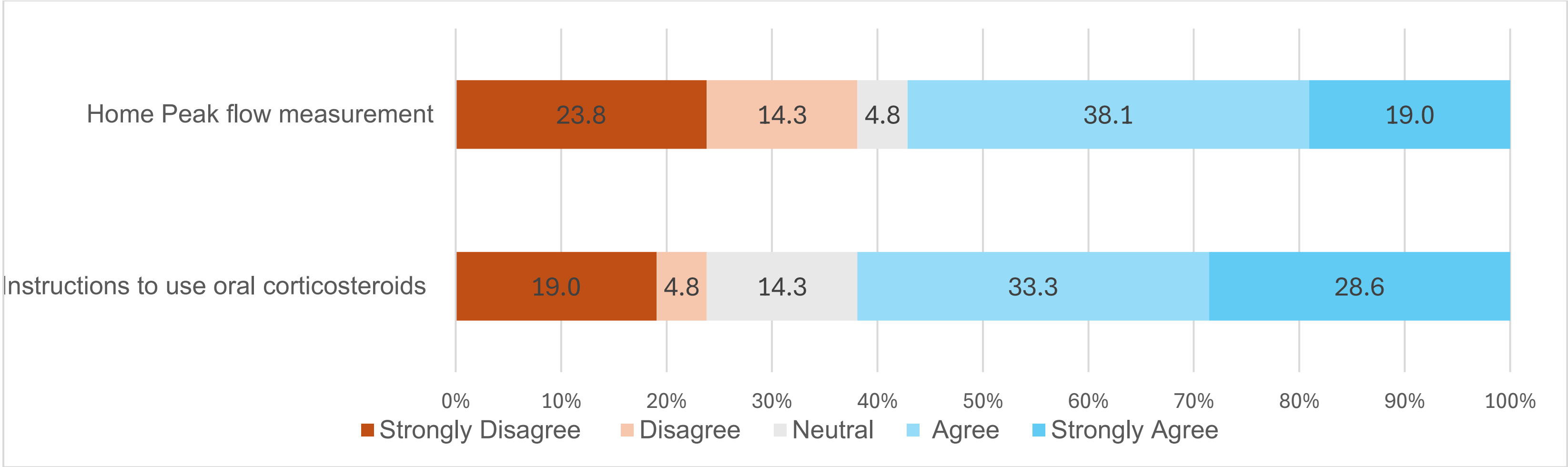

No agreement

No agreement

Section C – Severity Categorization

3. Q9: Would you agree that if a patient has these following signs and symptoms, he or she should be classified in the **green zone** of an asthma action plan?

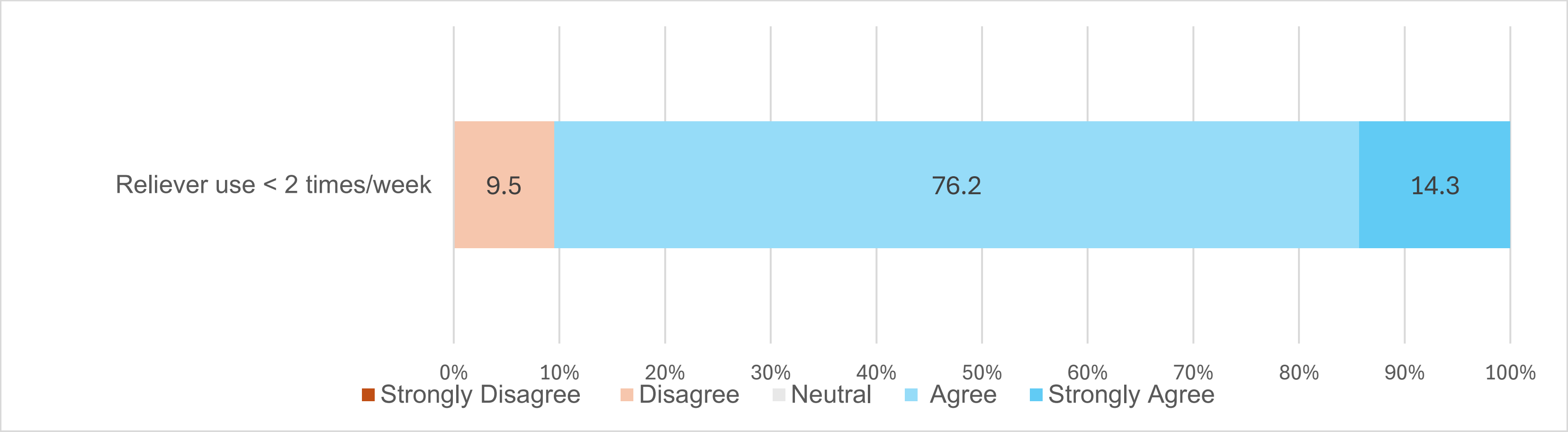

Agreement

4. Q13: Would you agree that each of the following signs or symptoms is as a “danger sign” that indicates the need for emergency care in an asthma action plan?

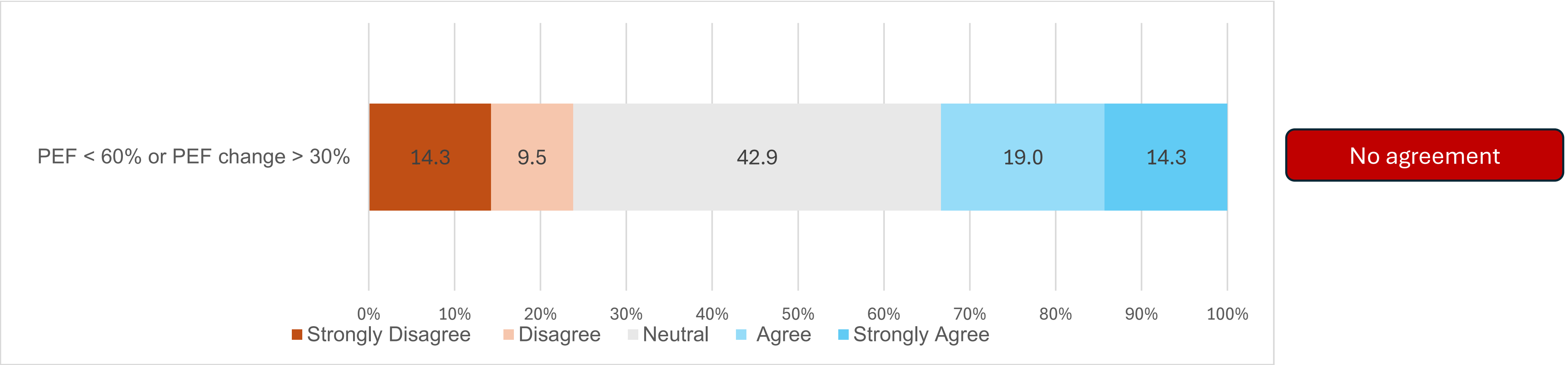

Section D – Recommended Actions for Each Zone/Symptom Severity

5. Q15: Would you agree that the following patient actions should be included in the action plan for Patients on MART (Formoterol + ICS combination) to ensure the appropriateness and feasibility in Vietnam settings?

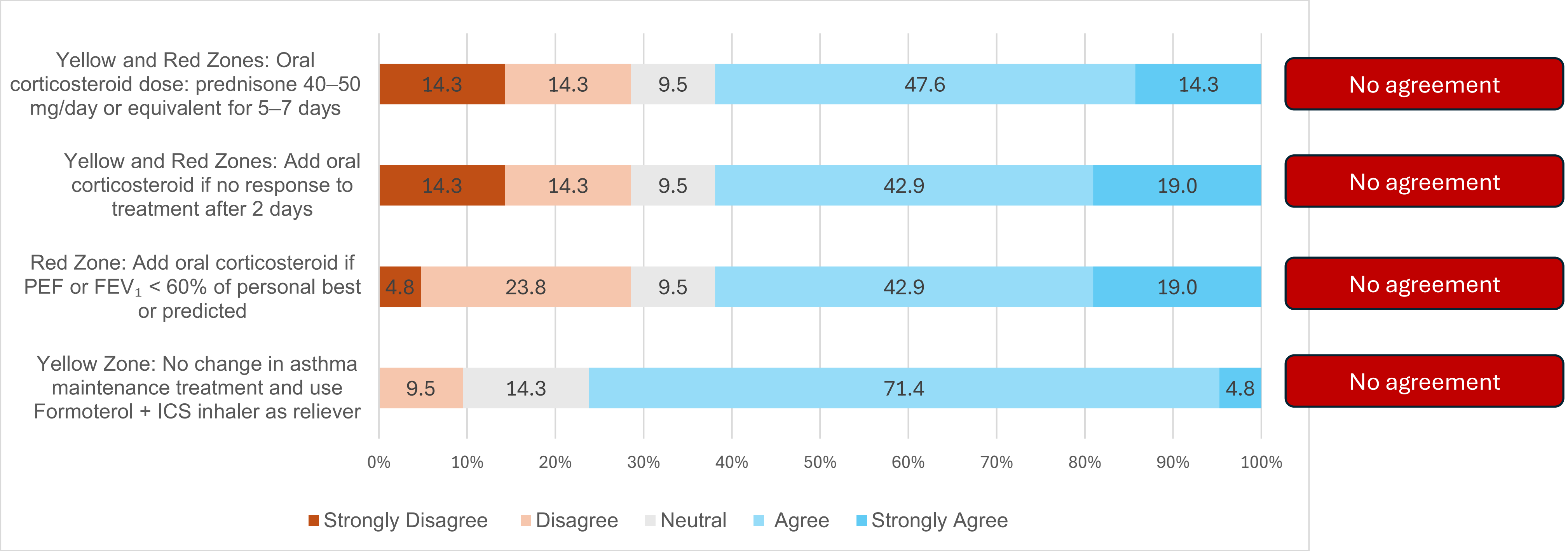

6. Q16: Would you agree that the following patient actions should be included in the action plan for Patients on ICS/LABA + SABA to ensure the appropriateness and feasibility in Vietnam settings?

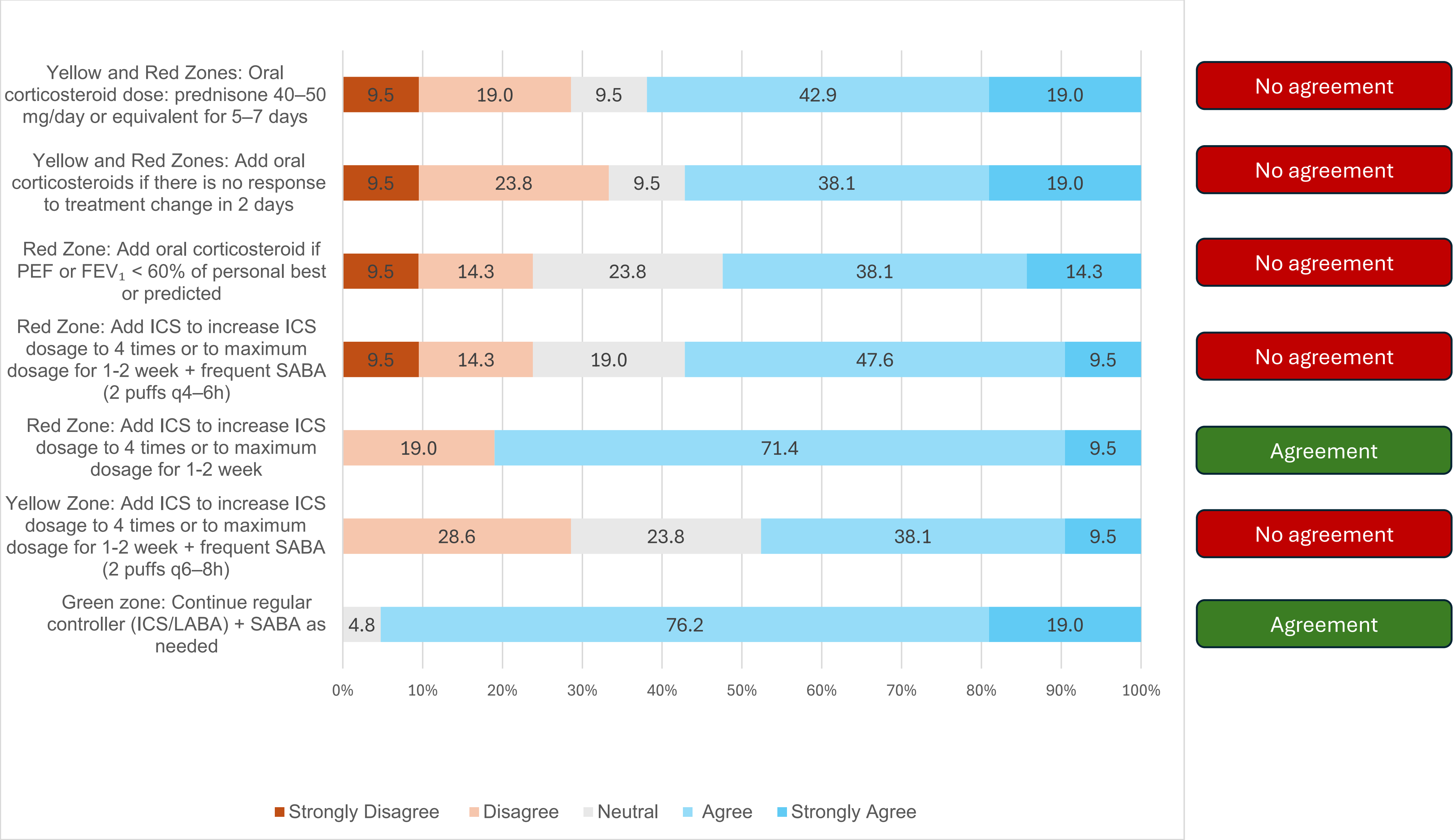

Supplement: Supplementary file 1 [file jcm-14-08640-s001.zip › jcm-4005389-supplementary.pdf]
